# Precocious Puberty and Covid-19 Into Perspective: Potential Increased Frequency, Possible Causes, and a Potential Emergency to Be Addressed

**DOI:** 10.3389/fped.2021.734899

**Published:** 2021-09-20

**Authors:** Maria E. Street, Chiara Sartori, Cecilia Catellani, Beatrice Righi

**Affiliations:** ^1^Division of Pediatric Endocrinology and Diabetology, Unit of Pediatrics, Department of Mother and Child, Azienda USL-IRCCS di Reggio Emilia, Reggio Emilia, Italy; ^2^PhD Program in Clinical and Experimental Medicine, University of Modena and Reggio Emilia, Modena, Italy

**Keywords:** precocious puberty, COVID-19, rapidly progressive puberty, precocious menarche, catecholamines, BMI, electronic devices, melatonin

## Abstract

A significant increase in precocious puberty, rapidly progressive puberty and precocious menarche has been reported in Italy since the initial lockdown because of the pandemic, and this could represent a new emergency to be addressed during this pandemic. There is a need, therefore, for further understanding and research. Many causes could account for this. Initially, it was thought that the changes in life-style, in screen time, and sleeping habits could be the cause but if considered individually these are insufficient to explain this phenomenon. Likely, changes in central nervous mediators, and an increase in catecholamines could contribute as a trigger, however, these aspects are poorly studied and understood as well as the real perceptions of these children. Finally, staying more indoors has certainly exposed these children to specific contaminants working as endocrine disruptors which could also have had an effect. It would be of utmost importance to compare this phenomenon worldwide with appropriate studies in order to verify what is happening, and gain a new insight into the consequences of the covid-19 pandemic and into precocious puberty and for future prevention.

## Introduction

Since mid 2020 several centers of pediatric endocrinology in Italy, including ours, have observed a significant increase in the number of female patients presenting with precocious puberty, precocious menarche, and rapidly progressing puberty. Until now these observations have been reported by the Meyer Hospital in Florence just after the lockdown (1), and very recently by the main pediatric hospital in Rome in a letter to the Editor (2). The first report observed an increase in the number of diagnoses and an acceleration in the progression of puberty, and hypothesized that this phenomenon was associated with the increased use of electronic devices and increased body mass index (BMI) subsequent to reduced physical activity and possibly increased eating. The second report confirmed the increase in the number of diagnoses of precocious puberty, and confirmed the net prevalence in females.

Many causes could be put forward but there is an urge for further understanding, and rapid preparation of research studies that need to study, and verify whether this phenomenon is confirmed, and its causes. Certainly, the possibility of comparing data with other Countries outside Italy that have dealt differently with the pandemic would be of great help. This phenomenon, at least in Italy, seems to be real in our experience too, and to be related with the persistent changes in lifestyle, school time and mode, and not with the March-April 2020 weeks of the initial lockdown only although thorough studies and reports are warranted.

**Figure 1:**
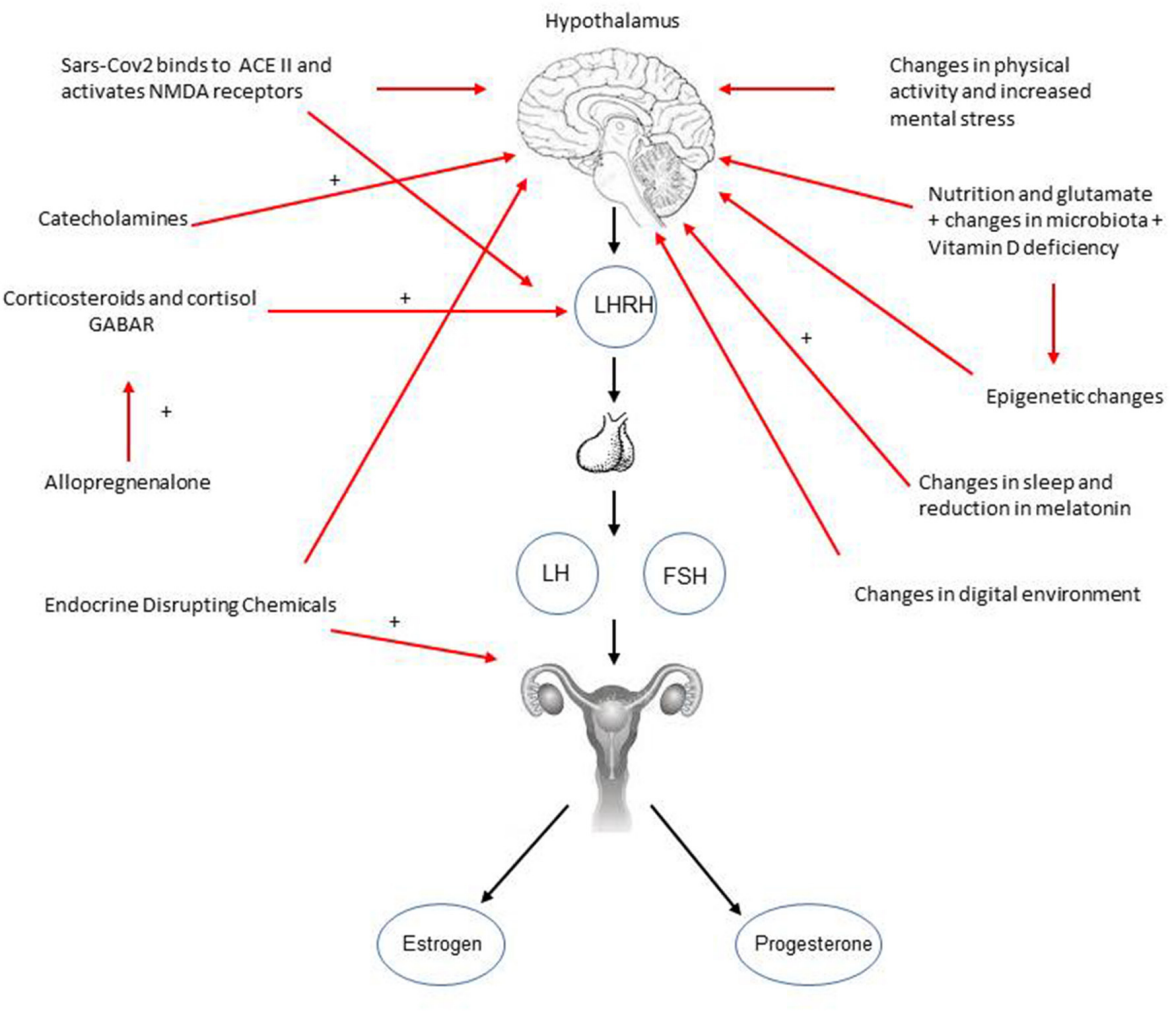
Possible factors contributing/causing precocious puberty and rapidly progressive puberty.

In the following paragraphs many aspects, potentially related with precocious puberty under these circumstances, will be further analyzed as the possible causes. A synthesis of these causes and the processes that could be involved in triggering and accelerating puberty are summarized in Figure 1.

## Possible Direct Effects of the SARS-COV2 Infection

First, a possible direct effect of the Sars-cov2 infection should be considered. Sars-Cov2 binds ACE-II receptors that are particularly abundant in the central nervous system, with subsequent neuronal transport, haematogenous and nasal route dissemination via the olfactory bulb, thus explaining one of the typical symptoms of covid-19 that is anosmia (3). The olfactory bulb is of great importance for chemical communication between the environment and the brain, and studies have shown inflammation of the olphactory bulb and epithelium related with covid-19 (4). Interestingly, an increased volume of the olphactory bulb has been described to be strongly correlated with precocious puberty onset (5). Gonadotropin Releasing Hormone (GnRH) neurons, which activation is necessary for the onset of puberty, share a common embryonic origin with the olphactory bulb neurons in this region. In addition, gamma-amino-butyrric (GABA) ergic neurons are also very abundant in this area, and are required for the control of the timing of puberty (6); therefore, a direct action of the virus on both these neurons is feasible, and could potentially trigger precocious puberty. The effects of Sars-cov2 on the central nervous system could occur through different mechanisms such as transportation through the blood-brain barrier, creating areas of increased permeability due to the infection, and through the activation of neural pathways. Furthermore, the increase in pro-inflammatory cytokines, specifically Interleukin (IL)-1 beta, IL-6 and tumor necrosis factor (TNF)-alpha, has an effect on the plasticity of synapsis, on neurogenesis, and on mechanisms of memory and learning (7). Interestingly, it has been shown that Sars-cov2 can activate N-Methyl-D-aspartate (NMDA) receptors (7) which promote pulsatile GnRH secretion and can accelerate puberty onset in rats (8). This could further suggest a possible direct effect of the virus infection on triggering puberty.

However, in our personal experience very few patients with recent diagnosis of precocious puberty had positive molecular diagnoses for Sars-cov2 infection, and most had a negative history and negative diagnostic tests in the previous months, and moreover, hypo/anosmia is rare in childhood. Altogether, based on these considerations the hypothesis of a direct effect of Sars-Cov2 as a trigger of puberty remains unlikely.

## Emotional and Psychological Factors

The considerations above lead to consider whether other factors, such as stress and fear of the risk of infection could have played a role because of their effect on specific central nervous system mediators. The Literature is currently lacking studies on these aspects in relationship with precocious puberty and the current pandemic. However, interestingly, in rats, increased corticosterone levels were shown to be able to modify the expression of the NR1, NR2A and NR2B subunits of NMDA neurons, contributing to explain the changes in mood that characterize puberty, and providing evidence also for the increased predisposition for mental illness in this period of life (8–10). These experimental conditions can be considered similar to a state of chronic stress, similar to that present since the beginning of the pandemic. It has been shown in female rats that in response to prolonged restraint stress, which is recognized as a feature of early puberty, a mediator of NMDA neuronal action, growth regulating factor 1 (GRF1), could regulate the hypothalamic pituitary axis besides regulating stress-induced plasticity of corticotropin releasing factor (CRF) cells (10). Another known factor contributing to stress-triggered anxiety at puberty is the activation of the GABA A receptors (GABAR), which are well known to play a pivotal role in anxiety besides in puberty (11). In female mice, this receptor is sensitive to allopregnanolone that has been previously shown in humans to be increased at onset of central precocious puberty (12). It could be then speculated that the precocity observed in girls could be related with a similar mechanism subsequent to the life conditions during this pandemic. Both allopregnanolone and cortisol should be studied in the female patients with precocious puberty presenting at our clinics during this pandemic.

Psychologists have correctly defined the pandemic as “a biological disaster with a subsequent strong psychological impact” (13). Increased catecholamines (dopamine and norepinephrine-NE) can also accelerate puberty; this has been shown in a female mouse model where both basal and potassium stimulated NE, after treatment with a partially purified male urine extract, was found to be increased in the posterior part of the olfactory bulb (14). Moreover, one study, in humans, reported increased precocious puberty after the use of methylphenidate, currently used for the treatment of attention deficit hyperactivity disorder, which blocks dopamine and NE transporters increasing their concentration in synaptic gaps (15). These could be capable of triggering puberty, and although these data still need to be confirmed, they might represent indirect evidence of a possible effect of catecholamines that needs to be further studied. It is currently unknown how much these mechanisms could be really of importance in humans.

The effect of stress within households has not been specifically addressed either by current studies, and may differ between Countries depending on differences in the adoption of restrictions to fight the pandemic. Families in Italy have often been forced to live in relatively small homes facing “distance learning”, “smart working”, and “financial problems” because of a reduction or loss of a regular income for long periods of time. It remains, therefore, to be elucidated whether these aspects could have had or have an effect on the timing of puberty.

## Nutrition, Adiposity, and Physical Activity

The pandemic has been characterized also by some changes in eating habits that need to be analyzed. Endogenous glutamate, as other aminoacids, stimulate GnRH secretion and NMDA receptors in immature mammals inducing precocious puberty (16). In particular, animal models have shown how glutamate and its NMDA subtype receptor regulate sexual maturation, and how daily injections of NMDA induce precocious puberty (17). At present it is unknown, however, whether an increased dietary uptake of glutamate and other aminoacids could accelerate puberty in humans, and this could represent a research topic to be pursued in the near future.

Nutrition is a known driver of sexual maturation (18). In malnourished adopted children a significant weight increase is well known to trigger precocious puberty (19–21), however, although during the pandemic BMI has generally increased because of reduced physical activity often this increase is not so significant (1, 2), and above all, none of these girls were malnourished before the pandemic. Therefore, it is unknown whether a modest increase in BMI alone could explain an increased frequency of cases of precocious puberty observed in clinics. A further consideration is that if the hypotheses above are correct, considering psychological distress, and the changes in diet including overeating during the pandemic, changes in DNA methylation, in the microRNA network and in the microbiota must also be taken into account. If this were the case in the next years these changes would determine an increase in ovarian dysfunction as polycystic ovary syndrome too (22, 23) that would need to be carefully monitored. Related with the current above aspects are reports of early puberty in girls born with a low birth weight but with subsequent rapid catch-up growth and subsequent increase in visceral fat as they often present an acceleration of puberty and an increased risk of polycystic ovary syndrome (24).

These may be important contributing factors, and changes have been reported internationally with regard to physical exercise practice, habits, pain, anxiety and stress with greater changes being observed in Italy (25). Eating habits were carefully evaluated in a German study showing mainly an increase in food amount, and specifically in the assumption of bread and confectionary, and changes in alcohol consumption were also reported. However, in a smaller group of people reduced food consumption was also described, and increased mental stress and sometimes increased physical activity were also referred (26). During the first lockdown in Italy, physical activity, and specifically walking and cycling, declined dramatically because of the features of the lockdown per se (27), and this clearly contributed further to explain a general increase in BMI.

## Changes In Sleep Habits

A further Italian study reported that students and workers presented also a worsening of sleep quality alongside an increased BMI (28). Sleep could be of importance as melatonin is related with sleep quality, and some studies have reported reduced melatonin serum concentrations in subjects with precocious puberty (29), and in physiological conditions at onset of puberty (30), thus suggesting a role for the pineal gland in regulating puberty besides sleep and melatonin secretion. Interestingly, melatonin is also involved with metabolism: meals taken at night instead of the correct time, when melatonin is high, lead to insulin resistance (31, 32). Moreover, in humans it has been reported that glucose tolerance was influenced by melatonin endogenous circadian rhythm (33) suggesting that changes in sleep and diet habits could have an effect on the timing of puberty. It is currently unknown, however, whether any of these changes could have been so significant to affect timing and progression of puberty in children.

## Changes In the Use of Digital Devices

Changes in neurotransmitters, in particular in serotonin and dopamine, have been described as a consequence of increased exposure to digital device environments with subsequent reduced amount of exposure to day light and sunlight (34). The pandemic has led to an overall increase in screen time both for recreational purposes (35), and school activities. All the changes in screen time, quality of sleep, and physical activity have been clearly reported for the students in the Guangzhou region in China where covid-19 was first reported (36). Whether these changes could have effects on the timing and progression of puberty, is currently unknown as the Literature is extremely scarce on this topic.

## Vitamin D Deficiency and Changes in the Exposure to Endocrine Disrupting Chemicals

Finally, the increase in BMI due to the amount in sedentary lifestyle, has paralleled an increased in indoor life which is associated also with vitamin D deficiency because of the reduced exposure to sunlight (37). Some authors found that vitamin D-deficient subjects are more likely to develop PP (38) so one might consider that this could potentially be an additional pre-existing contributing factor as it is unlikely that vitamin D levels can have changed significantly over this period of time. In addition, supplementation with vitamin D is recommended for fighting critical illness as can occur in covid-19 (39).

Finally, one must consider the possible effects of endocrine disrupting chemicals (EDC), such as polybrominated flame retardants, phthalate esters and bisphenol A used as plasticizers in many household objects (40–42), as they act as estrogen agonists and/or testosterone antagonists. The change in habits could have led to a major exposure to indoor contaminants which could have further contributed to the changes observed in the timing and progression of puberty (43).

## Discussion

Putting into perspective all the considerations above suggests that the pandemic could offer a unique opportunity to study the possible environmental triggers of puberty, and possibly to identify whether there is one major trigger or whether there is a combination of factors. Furthermore, one must consider that at present we need studies designed to verify:

- whether there is an increased frequency of precocious puberty, precocious menarche, rapidly progressive puberty Nationwide and in Europe, and to compare differences based on the different approaches adopted to face the pandemic, in particular in schools;
- the prevalence of covid-19 in the patients with the diagnoses above;
- any possible associations between all the above mentioned diagnoses and hormones (i.e. cortisol, allopregnanolone, catecholamines), and vitamin D serum concentrations, stress measurements by means of validated questionnaires, changes in sleep habits, melatonin serum concentrations, and any relationships with features of the pineal gland assessed by MRI scans.
- any possible associations between all the above mentioned diagnoses and life habits taking into account the time and mode of use of electronic devices, changes in diet, physical activity, BMI, changes in time and type of exposure to possible endocrine disruptors and other environmental contaminants considering the differences between industrialized and rural areas;
- how this phenomenon will evolve, if confirmed, once the pandemic is over and life turns to “regular” \ “normal” habits.

These studies are warranted to explore whether this phenomenon is confirmed, and in this case to find the causes of the important changes observed in Italy in the timing of puberty and in the time of progression of puberty. This would allow to put in place for the future preventive actions for this ongoing phenomenon and to give advice for future pandemic plans. Furthermore, one needs to study whether the increased frequency of precocious puberty and menarche, and rapidly progressive puberty have psychological consequences and/or effects on final height, body composition, and subsequent quality of life.

Finally, until now the increase in precocious puberty has been observed and reported in Italy in females only. It is yet unknown, but an effect in males cannot be excluded.

## Author Contributions

MS, CS, CC, and BR: conceptualization, writing—original draft preparation, and writing—review & editing. MS: visualization and supervision. All authors contributed to the article and approved the submitted version.

## Conflict of Interest

The authors declare that the research was conducted in the absence of any commercial or financial relationships that could be construed as a potential conflict of interest.

## Publisher's Note

All claims expressed in this article are solely those of the authors and do not necessarily represent those of their affiliated organizations, or those of the publisher, the editors and the reviewers. Any product that may be evaluated in this article, or claim that may be made by its manufacturer, is not guaranteed or endorsed by the publisher.
